# Elucidating the (lipo)Phenolic Composition of Olive Oil By‐Products and Biomass From Three Different *Olea Europaea* Cultivars by Liquid Chromatography Coupled to Photodiode Array and Mass Spectrometry

**DOI:** 10.1002/jssc.70189

**Published:** 2025-06-10

**Authors:** Francesco Cacciola, Wadir Mario Valentino Marchesiello, Micaela Galletta, Carmelo Coppolino, Marina Russo, Antonella Buccafuri, Alessia Fazio, Pierluigi Plastina, Maurizio Quinto, Paola Dugo, Luigi Mondello

**Affiliations:** ^1^ Messina Institute of Technology c/o Department of Chemical Biological, Pharmaceutical and Environmental Sciences, Former Veterinary School University of Messina Messina Italy; ^2^ Department of Agriculture Food, Natural Resources, and Engineering (DAFNE) University of Foggia Foggia Italy; ^3^ Department of Pharmacy Health and Nutritional Sciences University of Calabria Arcavacata di Rende CS Italy; ^4^ Chromaleont s.r.l., C/O Department of Chemical Biological, Pharmaceutical and Environmental Sciences Former Veterinary School University of Messina Messina Italy

**Keywords:** lipophenols, mass spectrometry, olive leaves, olive mill wastewater, olive pomace, phenols, RP‐HPLC

## Abstract

The olive oil industry is currently experiencing a period of significant growth, largely as a result of the high value placed on olive oil as a high‐nutritional food product on a global scale. Industrial olive oil production generates significant quantities of solid waste and dark liquid effluents, involving olive leaves (OLs), olive pomace (OP), and olive mill wastewaters (OMWWs). Particular attention was given to the recycling and valorization of by‐products and biomass from the olive oil industry due to their richness in bioactive compounds, including phenolic compounds with antioxidant properties. The aim of this study was the sustainable recovery and characterization of valuable biocompounds, notably phenols, with particular emphasis on lipophenols, from different matrices (OMWWs, OP, and OLs) of three different *Olea europaea* cultivars using LC coupled with photodiode array (PDA) and MS detection. Method validation demonstrated satisfactory linearity (*R* ^2^ > 0.9806), repeatability (lower than 9.82%) and recovery (over 79.07%) for all samples investigated. Before quantification, the matrix effect was also evaluated with values ranging from 71.99% to 139.70%.The results highlight that OLs, OP, and OMWWs might be employed for several applications, including nutraceuticals, cosmeceuticals, food, and animal feed.

## 1 Introduction

In the context of the food industry, the term “circularity” refers to the minimization of waste and the reuse of by‐products through the upgrading of these materials into co‐products. Particular emphasis was devoted to the implementation of effective sustainability practices, particularly within the oil industry, which is responsible for the generation of a considerable proportion of waste in the agri‐food sector, approximately 20% [1]. The olive oil industry represents a significant economic sector in the Mediterranean region. Global olive oil production reached approximately 3.2 million tons in 2022.

The leading olive oil‐producing countries are Spain, Italy, Greece, Turkey, and Tunisia, with Spain dominating the market, accounting for 40%–45% of global production. Specifically, Italy is the second largest producer in the world, producing around 315 000 tons [2]. The main producing regions are Apulia, which accounts for about 40% of national production, followed by Calabria, Sicily, and Tuscany. The area under olive cultivation in Italy covers approximately 1.1 million hectares, with Apulia being the largest producing region [3].

Olive oil is a valuable product obtained by pressing drupes harvested from the olive tree (*Olea europaea*). Its chemical composition is influenced by genetic factors (*cultivar*), environmental conditions, and edaphological characteristics, including climatic factors [4]. Despite its high economic and nutritional value, olive oil production has a negative environmental impact, caused by the generation of significant amounts of liquid and solid residues. Indeed, the Mediterranean countries collectively accumulate roughly 30 million m^3^ of olive mill waste each year [5]. Specifically, the solid waste consists of thin branches (approximately 50% by weight), olive leaves (OLs) (approximately 25% from the defoliators), and thick branches or wood (25%) [6]. After the extraction process, three different fractions are generated: olive oil (20%), olive pomace (OP) as the solid waste (30%), and olive mill wastewaters (OMWWs) as the liquid waste (50%). The primary challenge for the olive industry is the effective management of these residues, as they can, in certain cases, be a source of pollution and the costs associated with their proper disposal can be significant [7]. OP is a solid waste product that is composed primarily of water, pressed, and pulp. It is a rich source of organic molecules and contains high levels of potassium, low levels of phosphorus, and intermediate levels of nitrogen. Furthermore, it contains lipids [3, 8, 9]. OMWWs are the principal liquid waste product of olive oil production. It is characterized by a dark color and high concentrations of organic molecules, potassium, nitrogen, phosphorus, calcium, magnesium, and iron. Its composition depends on several factors, including the fertilizers employed, the water content of the olives, the climatic conditions, the storage and ripening conditions, and the chemical composition of the oil [10, 11, 12, 13]. Then, OLs are naturally shed during harvesting or results from regular tree pruning. Typically, they are used in direct combustion, serving as a source of fresh animal feed and as a resource for direct combustion or pellet production [14]. Due to the high content of phenolic bioactive compounds, OLs have been mainly exploited for the extraction of phenolic compounds [15, 16]. In particular, phenolic compounds, which are plant secondary metabolites, encompass a variety of different types, including flavonoids, isoflavonoids, phenolic acids, proanthocyanidins, tannins, and lignans [17]. These compounds are renowned for their biological properties and potential health benefits, including antimicrobial, antioxidant, anti‐inflammatory, and anti‐cancer activities. The potential of by‐products and biomass as effective sources of known phenolics with antioxidant properties has been demonstrated [18]. Therefore, these compounds are employed in the prevention of diseases associated with oxidative stress, including inflammatory disorders, diabetes, Alzheimer's disease, cancer, and cardiovascular disease [1, 7, 8, 9, 19, 20, 21]. The phenolic compounds in olive oil, ranging from 50 to 1000 mg kg^−1^, are simple phenols like phenolic acids and phenolic alcohols (hydroxytyrosol and tyrosol), polyphenols (flavonoids, apigenin, and luteolin), lignans (pinoresinol and 1‐acetoxypinoresinol), secoiridoids (oleuropein, glycosylated oleuropein, demethyloleuropein, elenolic acid, and ligstroside), and their aglycones [22]. Lipophenols, a subclass of phenols with a lipid component, have been demonstrated to preserve and, in some cases, enhance the antioxidant properties of their phenolic counterparts. Notably, homovanillyl oleate (HvOle) has been shown to possess significant radical scavenging and antioxidant properties [23], which help to reduce oxidative stress and may therefore aid in preventing or reducing skin aging. Furthermore, hydroxytyrosyl oleate (HtyOle) has been demonstrated to reduce nitric oxide (NO) production in a concentration‐dependent manner, suggesting potential immune‐modulating benefits. The presence of lipophenols or their precursors in olive oil and related by‐products is likely to be influenced by several factors including *cultivars*, ripening stage, geographical origin, and production procedures. It seems probable that these factors will affect the formation and distribution of these compounds among the different matrices, with the relative solubility in each matrix playing a role in this process [24, 25].

The bioeconomy involves the conversion of waste streams into valuable feedstocks using by‐products and biomass as renewable biological resources for the production of food, feed, bio‐based products, and bioenergy. After the olive oil extraction process, approximately 98%–99% of the phenolic compounds present in the drupe remain in the OP, which is rich in hydroxytyrosol, tyrosol, oleuropein, verbacoside and luteolin glycosides [26, 27]. OMWWs contain several phenolic compounds including hydroxytyrosol, tyrosol, verbascoside, and various acids such as caffeic, gallic, vanillic, and syringic. Polymeric substances are also present [10, 11, 28]. Phenolic extracts from OMWWs and OP can be used as natural alternatives to commercial synthetic antioxidants with applications in food as well as in the development of nutraceutical and medical products [29]. Both matrices have a high pollution index due to their acidic nature and high concentrations of salts and phenolic compounds, making them potentially harmful by‐products for the environment. Improper disposal of these by‐products could have serious environmental consequences as they contain organic compounds, which are potentially dangerous for soil and groundwater [27, 30]. Finally, the most abundant bioactive compounds in OLs are represented by oleuropein, tyrosol, and hydroxytyrosol [31].

Several researchers have reported the use of RP‐LC with UV and MS detection as the most adopted analytical tool for the phenolic characterization of *Olea europaea* [32, 33, 34, 35, 36, 37, 38]. Among the available stationary phases, conventional C18 columns have been the most commonly employed [33, 35, 36]; notably, stationary phases based on pentafluorophenyl (PFP) have also been successfully used for phenols separation due to their slightly higher polarity and unique separation mechanism [32, 38]. Specifically, PFP phases enable π–π interactions with the aromatic rings of phenols [37], which result in improved resolution and separation efficiency for this class of compounds [39]. However, all these works have dealt with the phenolic composition of olive oil by‐products and biomass, and no one comprehensively has focused on the lipophenolic fraction. Only HvOle, HtyOle, and tyrosyl oleate (TyOle) have been individually identified by LC coupled with MS [23, 25, 40]. Further, the proliferative properties of TyOle on human keratinocytes (HaCat) were evaluated by 3‐(4,5‐dimethylthiasol‐2‐yl)‐2,5‐diphenyltetrazolium bromide (MTT) assay [40]. In such a context, the present research is focused on the distribution and determination of the bioactive molecules (phenols and lipophenols) from liquid (OMWWs) and solid/semisolid by‐products and biomass (OP and OLs, respectively).

Given the above, the phenolic and lipophenolic factions of OMWWs, OP, and OLs were characterized by LC using a PFP column coupled with a photodiode array (PDA) and MS detection.

## 2 Materials and Methods

### 2.1 Chemicals and Reagents

Oleacin (≥ 90%), oleocanthal (≥ 95%), oleuropein (≥ 98%), verbascoside (≥ 99%), hesperidin (≥ 80%), naringin (≥ 95%), apigenin (≥ 99%), hydroxytyrosol (≥ 90%), tyrosol (≥ 95%), and eriocitrin (≥ 98%) reference standard materials, as well as homovanillyl alcohol, methyl oleate, and methyl linoleate, were purchased from Merck Life Science (Merck KGaA, Darmstadt, Germany). Tyrosyl oleate (TyOle), hydroxytyrosyl oleate (HtyOle), and homovanillyl oleate (HvOle) were previously synthesized in our laboratory [23, 25, 40]. The synthesis of tyrosyl linoleate (TyLi), hydroxytyrosyl linoleate (HtyLi), and homovanillyl linoleate (HvLi) was carried out expanding the scope of this method to the use of methyl linoleate instead of methyl oleate. This enzymatic approach is based on the use of *Candida antarctica* Lipase B (CALB) immobilized on Immobead 150, obtained from Novozymes (Bagsværd, Denmark). Each phenolic (tyrosol, hydroxytyrosol, or homovanillyl alcohol, 1.6 mmol) was reacted with methyl linoleate (3.2 mmol) at 50°C for 48 h in an orbital shaker, using CALB and *t*‐butanol (2 mL) as the catalyst and the solvent, respectively. The mixture was cooled down, the enzyme was filtered off, and the solvent was evaporated at reduced pressure. The purification of lipophenols was attained by column chromatography (SiO_2_, eluent: *n*‐hexane/acetone). Acetonitrile, water, *n*‐hexane, ethanol, methanol, acetone, and formic acid were all HPLC grade and were provided by Merck Life Science (Merck KGaA, Darmstadt, Germany).

### 2.2 Sample Collection

The present study was carried out on samples of OMWWs, OP, and OLs collected in November 2023 right after the milling process. The samples were derived from three distinct olive cultivars (*Roggianella*, *Nocellara*, and *Coratina*) provided by three local olive oil producers in Cosenza (Calabria, Italy), Messina (Sicily, Italy), and Bari (Apulia, Italy), respectively. For all cultivars the milling process was carried out with closed malaxers, at these conditions: 35°C for 50–85 min with an addition from 22% to 28% of water.

### 2.3 Phenolic Compounds Extraction

All samples underwent liquid‐liquid or solid‐liquid extraction of phenolic compounds before analysis. The extraction of these compounds from OMWWs, OP, and OLs was carried out following the previously optimized procedure established by our research group with certain modifications [38]. Briefly, 1 mL of OMWWs was extracted with 1 mL of ethyl acetate through sonication for 15 min, followed by sample centrifugation for 10 min at 4500 RPM, using a Neya XS centrifuge (REMI Sales & Engineering Ltd., Maharashtra, India) to separate different phases. The extraction process was performed three times using the same volume of solvent. The three ethylacetate phases were then collected and dried by using a rotary evaporator. The final extract was then dissolved in 1 mL of methanol and filtered through a membrane filter with a pore size of 0.45 µm.

On the other hand, a solid‐liquid extraction was carried out on OP and OLs. Specifically, 10 g of OP was weighed, and added to 10 mL of *n*‐hexane three times and the hexane phase was discarded (only for phenols extraction). Then, 1 g of the OP residue was extracted with 3 mL of methanol and sonicated for 15 min. Four aliquots of the extract were collected and dried with a rotary evaporator. The final extract was then dissolved in 1 mL of methanol before LC‐PDA/MS analysis.

Approximately 2.5 g of OLs were grounded, weighed, and then extracted with 15 mL of a mixture of methanol:water (50:50; *v/v*). The procedure was repeated five times and the extract aliquots were collected and dried in an evaporator. The final extract was then dissolved in 1 mL of a mixture of methanol. Each sample was analyzed in triplicate.

### 2.4 RP‐LC‐PDA/MS Method

RP‐LC‐PDA/MS analyses were conducted on a Shimadzu Nexera LC‐30A system (Shimadzu, Kyoto, Italy), consisting of a CBM‐20A controller, two LC‐30AD dual‐plunger parallel‐flow pumps, a DGU‐20A5 degasser, a CTO‐20A oven, a SIL‐30AC autosampler, and a SPD‐M30A detector. The LC system was connected to a single‐quadrupole LCMS‐2020 mass spectrometer equipped with an ESI interface (Shimadzu, Kyoto, Japan). Separations were carried out on an Ascentis Express F5 (150 × 4.6 mm, 2.7 µm) analytical column (Merck Life Science, Merck KGaA, Darmstadt, Germany). The employed mobile phases were water with 0.1% formic acid (A) and acetonitrile with 0.1% formic acid (B), and the elution gradient was set as follows: 5 min 5% B, 15 min 30% B, 40 min 60% B, 47.50 min 80% B, 50 min 100% B, 55 min 100%, 58 min 5%, 65 min 5%. The flow rate was 1 mL min^−1^, the oven temperature was 30°C, and the injection volume was 5 µL. The following MS parameters, through the ESI source in negative (−) ionization mode, were employed: the interface, desolvation line (DL), and heat block temperatures were set at 350°C, 250°C, and 400°C, respectively; nebulizing gas and drying gas flow (N_2_) were set at 1.5 L min^−1^ and 10 L min^−1^, respectively; and the acquisition MS range was 100–700 *m/z* with an event time of 1 s. The DL voltage and Qrayy DC voltage were set at the default value, and the detector voltage was set at −1.20 kV. Single ion monitoring mode (SIM) was used for phenols and lipophenols quantification: tyrosol (137 *m/z*), hydroxytyrosol (153 *m/z*), apigenin (269 *m/z*), oleacein (319 *m/z*), oleuropein (539 *m/z*), verbascoside (624 *m/z*), hydroxytyrosyl oleate (417 *m/z*), tyrosyl oleate (401 *m/z*), homovanillyl oleate (431 *m/z*), hydroxytyrosyl linoleate (415 *m/z*), tyrosyl linoleate (399 *m/z*), and homovanillyl linoleate (429 *m/z*). Furthermore, for the extraction method validation eriocitrin (595 *m/z*), hesperidin (609 *m/z*), and naringin (579 *m/z*) were monitored. Data acquisition was performed by using Lab Solution v. 5.97 software (Shimadzu, Kyoto, Japan).

### 2.5 HPLC Method Validation

A stock solution of 1000 mg L^−1^ of each reference standard material was prepared in different solvents: dimethyl sulfoxide for hydroxytyrosol and apigenin, dimethylformamide for tyrosol, oleuropein, and verbascoside, ethanol for oleacin and oleocanthal, and methanol for each synthesized lipophenolic compound. Mixture solutions were prepared by diluting each stock solution in a 1 mL flask with water:acetonitrile (95:5; *v/v*) and methanol, respectively. The standard solutions were stored at −20°C, brought back to room temperature, and sonicated for ten minutes before the injection into the RP‐LC‐PDA/MS system. Calibration curves were created in PDA and SIM mode for all the target analytes. Five replicates were carried out for each level. Several figures of merit were assessed: linearity, LoD and LoQ, precision (intra‐ and inter‐day), accuracy, matrix effect (ME), and recovery. Linearity parameters (i.e., slope, intercept significance, *R* ^2^) were evaluated through the regression study. The area of each standard compound was extrapolated, and the standard deviation (*s*) was calculated. The *s* ^′^ parameter was estimated, by dividing the *s* value with the slope. Thus, LoD and LoQ values were obtained by multiplying the *s* ^′^ of the analyte area at the lowest concentration level with a factor of 3 and 10, respectively. Intra‐ and inter‐day repeatabilities were expressed as coefficient of variation (CV %) of one concentration level (25 mg L^−1^). Five replicates were performed on the same day (*n* = 5) and over two consecutive days (*n* = 10), for intra‐ and inter‐day repeatability, respectively. Accuracy was calculated at 25 mg L^−1^ for each standard.

### 2.6 Extraction Method Validation

Considering the difficulty of finding a “blank” sample, the matrix was fortified with three different flavonoids (eriocitrin, hesperidin, and naringin), typically present in *Citrus* fruits, and not present in the analyzed samples [41]. The absence of these flavonoids was confirmed by preliminary analysis. A stock solution (100 mg L^−1^) of the three flavonoids was prepared in ethanol and diluted at different concentration levels between 5 and 20 mg L^−1^ for the calibration curve. The validation of the extraction method was then carried out on all the analysed matrices (OMWWs, OP, and OLs) spiked with a known amount of a mixture containing eriocitrin, hesperidin, and naringin (20 mg L^−1^). Each extract was analyzed in triplicate. The recovery was calculated according to the following formula:

$$\begin{array}{c} \mathrm{Recovery}\;\%\;=\frac{\begin{array}{c} \mathrm{Conc}.\;\mathrm{Standard}\;\mathrm{mixture}\;\mathrm{in}\;\mathrm{Pre}\\ -\;\mathrm{Fortified}\;\mathrm{extraction} \end{array}}{\begin{array}{c} \mathrm{Conc}.\;\mathrm{Standard}\;\mathrm{mixture}\;\mathrm{in}\;\mathrm{Post}\\ -\;\mathrm{Fortified}\;\mathrm{extraction} \end{array}}\\ \times 100 \end{array}$$

The ME, resulting from the loss or enrichment of the analyte during the extraction steps, was estimated at the same concentration level of the flavonoid mixture as the analyzed samples. The ME was calculated according to the following formula:

$$\begin{array}{ccc} \mathrm{ME}\;\%\; & = & \frac{\mathrm{Conc}.\;\mathrm{Standard}\;\mathrm{mixture}\;\mathrm{in}\;\mathrm{Post}-\mathrm{Fortified}\;\mathrm{extraction}}{\mathrm{Conc}.\;\mathrm{Standard}\;\mathrm{mixture}\;\mathrm{in}\;\mathrm{solvent}}\\ & & \times 100 \end{array}$$

## 3 Results and Discussion

### 3.1 RP‐HPLC‐ PDA/MS Method Validation

The objective of this research was to develop and validate a systematic analytical method suitable for the identification and quantification of (lipo)phenols in OMWWs, OP, and OLs. The initial step involved the optimization of the chromatographic conditions and ESI parameters. The event time was varied in the range of 0.5–1 s to examine the potential gain in signal intensity without the influence of the ion space‐charge effect. The MS analyses were performed in negative ion mode, which guaranteed higher sensitivity for each compound and clearer mass spectra. Chromatographic analysis was achieved by RP‐LC and MS detection. The PFP stationary phase was selected for its higher resolution and efficiency in phenol separation compared to other types of columns [39]. Moreover, it resulted in success for lypophenol separation. Table 1 shows the method validation results in terms of linearity, LoD and LoQ, intra‐ and interday, precision, and accuracy. Calibration curves were built in SIM mode for all standards dissolved in solvent. The linearity of the analytical procedure was found to be satisfactory for each target compound, with correlation coefficients ranging from 0.9768 to 0.9997. The intra‐ and interday CV % obtained were below 9.82% for inter‐day and 6.58% for intraday measurements, respectively, confirming the acceptability of the method.

**TABLE 1:** LoD and LoQ, linearity ranges, regression equations, correlation coefficient (*R* ^2^), intra‐ and inter‐day repeatability (expressed as CV %), and accuracy (expressed as %) values for each reference standard analyzed.

Compounds	[M‐H]^−1^	Linearity range (mg L^−1^)	Equation	*R* ^2^	LoD (mg L^−1^)	LoQ (mg L^−1^)	Repeatability (%) 25 mg L^−1^		Accuracy (%) 25 mg L^−1^
							intra‐day	inter‐day	
Hydroxytyrosol	153.2	1–500	*y* = 38944*x* + 220528	0.9826	0.004	0.012	1.55	9.82	110.12
Tyrosol	137.2	1–500	*y* = 2544*x* − 867	0.9993	0.036	0.121	5.82	5.57	87.83
Verbascoside	623.6	1–500	*y* = 264429*x* + 1282478	0.9876	0.057	0.189	1.45	5.71	95.08
Oleuropein	539.5	1–500	*y* = 381404*x* + 3305948	0.9873	0.041	0.138	2.92	2.89	113.45
Oleacein	319.3	1–500	*y* = 256606*x* + 768057	0.9946	0.003	0.011	4.95	4.79	106.59
Oleocanthal	303.3	1–500	*y* = 10851*x* + 16381	0.9975	0.093	0.311	3.45	9.34	102.40
Apigenin	269.0	1–500	*y* = 390854*x* + 8453689	0.9847	0.018	0.059	2.12	7.60	118.42
Hydroxytyrosyl linoleate	415.3	1–500	*y* = 184924*x* + 1676018	0.9806	0.083	0.276	3.35	6.35	97.51
Hydroxytyrosyl oleate	417.6	1–500	*y* = 149829*x* + 1629815	0.9867	0.062	0.205	4.81	7.21	103.22
Tyrosyl linoleate	399.3	1–500	*y* = 963*x* + 231	0.9997	0.118	0.393	4.25	8.32	105.59
Homovanillyl linoleate	429.3	1–500	*y* = 516*x* − 3497	0.9835	0.313	0.870	3.54	6.22	99.31
Tyrosyl oleate	401.6	1–500	*y* = 856*x* + 890	0.9977	0.001	0.002	6.58	2.51	100.20
Homovanillyl oleate	431.6	1–500	*y* = 696*x* − 6476	0.9932	0.375	0.951	2.11	5.8	102.31

### 3.2 Extraction Method Validation

All the analyzed samples were subjected to solvent extraction before RP‐LC analyses. Recovery was used to evaluate the effectiveness of these sample preparation procedures. In detail, each sample was fortified with a known amount (20 mg L^−1^) of eriocitrin, hesperidin, and naringin. Extraction recovery was calculated as described in the Materials and Methods section. The results, reported in Table 2, show that satisfactory recovery values (all above 79.07%) were obtained for the selected analytes, and specifically, values ranged from 79.07% to 123.05%.

**TABLE 2:** Recovery data (expressed as %) for eriocitrin, naringin and hesperidin in OMWWs, OP, and OLs samples.

Compounds	OMWWs			OP			OLs		
	*Roggianella cultivar*	*Coratina cultivar*	*Nocellara cultivar*	*Roggianella cultivar*	*Coratina cultivar*	*Nocellara cultivar*	*Roggianella cultivar*	*Coratina cultivar*	*Nocellara cultivar*
Eriocitrin	79.07 ± 0.48	82.31 ± 0.41	84.62 ± 0.35	102.56 ± 3.51	103.89 ± 6.43	115.22 ± 4.38	106.38 ± 10.31	117.17 ± 15.83	106.97 ± 15.13
Naringin	81.51 ± 2.29	90.41 ± 2.08	98.94 ± 2.54	107.68 ± 5.55	103.02 ± 13.37	115.88 ± 6.70	114.58 ± 10.77	119.61 ± 11.18	119.74 ± 13.34
Hesperidin	80.81 ± 0.30	96.35 ± 1.65	88.08 ± 0.41	105.21 ± 1.64	99.42 ± 8.64	110.29 ± 4.32	123.05 ± 12.93	122.53 ± 15.45	103.79 ± 16.42

Before quantification, the ME was evaluated by comparing the peak area at a given concentration, spiked after the extraction into the sample, to the MS response of the same analyte in the pure solvent. Results are reported in Table S1 and show values ranging from 71.99% to 139.70%. It is well established that a value of 100% implies a lack of ME; on the other side, a value higher than 100% means a signal enhancement, whereas a value lower than 100% exhibits a signal suppression. For both OMWWs and OP samples and all three cultivars, ion enhancement effects were obtained except for the hesperidin in the *Roggianella* cultivar. Specifically, OMWWs (*Nocellara* cultivar) turned out to be the most complex in terms of matrix interferences, along with the OP sample, belonging to the same cultivar. A peculiar behavior was attained for analytes in the OLs of all cultivars, which showed ME% values ranging from 71.99 to 92.13. This aspect may depend on the sample matrix to analyte concentration ratio [42].

### 3.3 Analysis of By‐Products and Biomass

**FIGURE 1:**
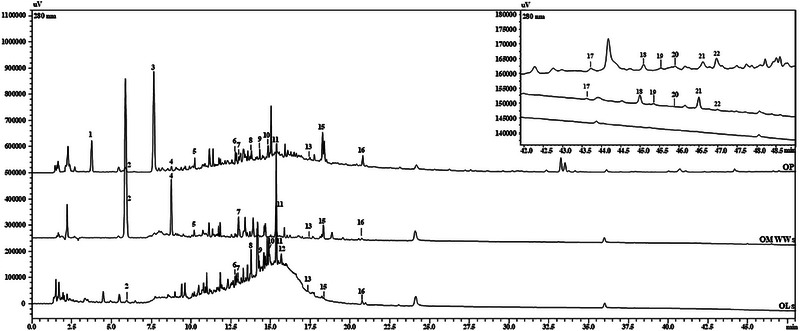
RP‐LC‐PDA chromatograms extracted at 280 nm of the OP, OMWWs, and OLs from *Coratina* cultivar. Compounds are referred to numbers, corresponding to those listed in Table 2.

The validated method was employed to characterize the different samples, and the quali‐quantitative results of the nine analyzed samples are reported in Figure 1 and Table 3. All samples displayed variable compositions and concentrations depending on the *cultivar*. OMWWs were found as the richest sources of polar phenolic compounds, in terms of the number of identified compounds, as well as their quantity. This may be due to the high solubility of these polar compounds in this aqueous matrix, in agreement with a previous study where a quantity as high as 432.1 mg g^−1^ was reported, compared to pomace, olives, and extra virgin olive oil (EVOO) [38]. *Coratina* and *Roggianella* OMWWs showed higher levels of hydroxytyrosol (259.21 ± 3.19 mg kg^−1^ and 202.5 ± 3.07 mg kg^−1^) and tyrosol (158.60 ± 0.68 mg kg^−1^ and 148.25 ± 2.07 mg kg^−1^) with respect to *Nocellara* one where values of 95.44 ± 2.32 mg kg^−1^ and 49.37 ± 0.19 mg kg^−1^ were obtained. Remarkable quantities of hydroxytyrosol were obtained also for the OL samples, especially for the cultivars *Roggianella* and *Nocellara* (86.59 ± 2.33 and 27.57 ± 1.43 mg kg^−1^), whereas values under the LoQ were found in all OP cultivars. Tyrosol was not detected in all OL cultivars. *Nocellara* OMWWs were characterized by the presence of a content of verbascoside as high as 25.32 ± 0.11 mg kg^−1^. Considering secoiridoids, oleacein was more abundant in *Nocellara* OMWWs (11.90 ± 1.31 mg kg^−1^), followed by the *Roggianella* OP (9.52 ± 0.32 mg kg^−1^), oleuropein was present in all samples of all cultivars, with *Nocellara* and *Coratina* OLs showing the maximum concentration of 55.28 ± 0.77 mg kg^−1,^ and 45.09 ± 0.54 mg kg^−1^ which is in line with literature studies [16]; notably, oleocanthal was absent in almost all samples and quantifiable only in the *Coratina* OP (1.57 ± 0.43 mg kg^−1^). With regards to flavonoids, two of them were identified and specifically luteolin and apigenin; the former was especially found in the OP sample of all cultivars (15.10 ± 0.70 mg kg^−1^ for *Roggianella*, 20.10 ± 0.89 mg kg^−1^ for *Coratina* and 11.10 ± 1.21 mg kg^−1^ for *Nocellara*), likewise the latter, with the highest amount of 15.21 ± 0.93 mg kg^−1^ determined in the *Coratina* OP.

**TABLE 3:** Concentration (mg kg^−1^ ± standard deviation) of (lipo)phenols determined in OMWWs, OP, and OLs samples.

		OMWWs			OP			OLs		
Peak No.	Compounds	*Roggianella cultivar*	*Coratina cultivar*	*Nocellara cultivar*	*Roggianella cultivar*	*Coratina cultivar*	*Nocellara cultivar*	*Roggianella cultivar*	*Coratina cultivar*	*Nocellara cultivar*
1	Gallic acid	2.59 ± 2.11	n.d.	n.d.	3.20 ± 1.65	5.17 ± 1.65	n.d.	7.51 ± 2.65	n.d.	n.d.
2	Hydroxytyrosol	202.50 ± 3.07	259.21 ± 3.19	95.44 ± 2.32	<LOQ	<LOQ	<LOQ	86.59 ± 2.33	16.19 ± 0.65	27.57 ± 1.43
3	Protocatechuic acid	8.57 ± .57	n.d.	4.52 ± 1.51	10.1 ± 1.83	25.52 ± 2.84	4.81 ± 3.11	11.1 ± 2.30	n.d.	5.71 ± 2.23
4	Tyrosol	148.25 ± 2.07	158.60 ± 0.68	49.37 ± 0.19	n.d.	8.07 ± 0.57	n.d.	n.d.	n.d.	n.d.
5	Caffeic acid	<LOQ	1.20 ± 1.12	0.89 ± 1.35	1.21 ± 2.25	2.51 ± 0.98	<LOQ	1.52 ± 1.12	n.d.	<LOQ
6	Luteolin 3′,7‐O‐diglucoside	<LOQ	n.d.	0.67 ± 3.21	0.89 ± 1.23	0.79 ± 1.94	<LOQ	0.57 ± 1.11	0.80 ± 3.47	<LOQ
7	Verbascoside	<LOQ	15.17 ± 0.17	25.32 ± 0.11	1.32 ± 0.34	<LOQ	<LOQ	<LOQ	<LOQ	<LOQ
8	Luteolin glucoside a	<LOQ	n.d.	<LOQ	0.52 ± 0.22	0.67 ± 0.90	0.89 ± 2.34	1.31 ± 4.22	2.10 ± 3.22	1.35 ± 2.22
9	Luteolin glucoside b	<LOQ	n.d.	<LOQ	0.31 ± 3.13	0.72 ± 0.84	0.21 ± 1.89	0.89 ± 1.58	1.81 ± 2.52	<LOQ
10	Luteolin glucoside c	<LOQ	n.d.	1.21 ± 3.36	0.21 ± 2.21	n.d.	0.18 ± 0.55	1.12 ± 1.11	5.79 ± 2.55	3.60 ± 2.55
11	Oleuropein	<LOQ	22.10 ± 0.55	<LOQ	2.07 ± 0.37	0.92 ± 2.44	<LOQ	9.08 ± 0.56	45.09 ± 0.54	55.28 ± 0.77
12	Luteolin glucoside d	<LOQ	n.d.	2.11 ± 2.25	1.89 ± 1.21	0.52 ± 3.52	0.99 ± 2.52	1.10 ± 0.37	1.22 ± 1.55	1.23 ± 1.44
13	Oleacein	<LOQ	0.26 ± 0.20	11.90 ± 1.31	9.52 ± 0.32	0.52 ± 0.39	7.72 ± 0.23	n.d.	1.94 ± 0.07	3.52 ± 0.08
14	Oleocanthal	<LOQ	n.d.	n.d.	n.d.	1.57 ± 0.43	n.d.	n.d.	n.d.	n.d.
15	Luteolin	<LOQ	3.55 ± 0.88	<LOQ	15.10 ± 0.70	20.10 ± 0.89	11.10 ± 1.21	<LOQ	<LOQ	<LOQ
16	Apigenin	1.52 ± 0.02	3.50 ± 0.22	<LOQ	6.50 ± 0.75	15.21 ± 0.93	1.51 ± 1.88	4.50 ± 2.22	<LOQ	1.92 ± 0.22
17	Hydroxytyrosyl linoleate	2.56 ± 0.02	3.37 ± 0.03	4.05 ± 0.02	n.d.	8.25 ± 0.29	n.d.	n.d.	n.d.	<LOQ
18	Hydroxytyrosyl oleate	3.81 ± 0.06	12.88 ± 0.28	5.64 ± 0.40	3.32 ± 0.18	15.22 ± 1.80	3.88 ± 0.01	n.d.	n.d.	n.d.
19	Tyrosyl linoleate	n.d.	1.33 ± 0.05	0.71 ± 0.02	n.d.	6.69 ± 0.67	n.d.	n.d.	n.d.	n.d.
20	Homovanillyl linoleate	n.d.	0.99 ± 0.01	2.36 ± 0.01	n.d.	1.97 ± 0.16	n.d.	n.d.	n.d.	n.d.
21	Tyrosyl oleate	<LOQ	17.71 ± 0.11	0.90 ± 0.05	3.00 ± 0.28	8.38 ± 2.21	3.10 ± 0.12	<LOQ	<LOQ	<LOQ
22	Homovanillyl oleate	n.d.	1.91 ± 0.07	n.d.	n.d.	4.37 ± 0.15	n.d.	n.d.	n.d.	n.d.

With regards to lipophenols, namely oleic and linoleic esters of tyrosol, hydroxytyrosol, and homovanillic alcohol, they have been only scatteredly reported and never comprehensively identified in any EVOO bio‐product. This class of chemicals is of particular interest since they are reported to preserve and, in some cases, increase the antioxidant activity of the corresponding phenol. Moreover, the increased lipophilicity given by the lipidic moiety increases the bioavailability of this compound class in terms of absorption and metabolic stability [23]. With the exception of OL samples, all of them were found in all the OMWWs and OP of the *Coratina* samples. Notably, HtyOle and TyOle were found as the major lipophenols in *Coratina* OMWWs (12.88 ± 0.28 mg kg^−1^ and 17.71 ± 0.11 mg kg^−1^) and OP (15.22 ± 1.80 mg kg^−1^ and 8.38 ± 2.21 mg kg^−1^). HvOle was found only in *Coratina* samples, in particular at the concentrations of 4.37 ± 0.15 mg kg^−1^ and 1.91 ± 0.07 mg kg^−1^ for OP and OMWWs, respectively. These lipophenols have been already identified and quantified in olive oil as well as in its industrial by‐products, in particular HtyOle in OP and OMWWs [25], TyOle in OP [40], and HvOle in OLs and OP [23]. However, in this work, linoleic esters of phenols have been identified for the first time in OMWWs and OP. Among them, HtyLi was the most abundant since it was found in all OMWWs and *Coratina* OP samples, with the latter showing the highest concentration of 8.25 ± 0.29 mg kg^−1^. TyLi was identified in two OMWWs and in *Coratina* OP samples, the latter with the highest amount of 6.69 ± 0.67 mg kg^−1^. HvLi was found in the same samples where TyLi was present. In particular, *Nocellara* OMWW samples showed the highest HvLy concentration (2.36 ± 0.01 mg kg^−1^). Compared to oleic esters, linoleic esters were found at lower concentrations, most likely because of the most abundant oleic acid in olive oil over linoleic acid.

## 4 Conclusions

This study represents an initial comprehensive analysis of (lipo)phenolic compounds present in by‐products, including OMWW, OP, and biomass such as olives and OLs, with a particular emphasis on the presence of the (lipo)phenolic compounds within these matrices. The primary aim was to identify qualitative and quantitative changes in (lipo)phenol content within these by‐products and biomass materials. Our findings indicate that these matrices contain substantial amounts of (lipo)phenolic compounds, especially OP is a valuable raw material, rich in highly beneficial compounds known for their significant health benefits and technological properties that could be employed for several applications, including nutraceuticals, cosmeceuticals, food, and animal feed.

Following the investigation of these molecules, future research should focus on their isolation from olive samples through preparative multidimensional liquid chromatography (prep‐MDLC). This technique which involves automated fraction collection, represents an optimal solution for purifying molecules in complex samples with high purity and yield [41].

## Author Contributions

**Francesco Cacciola**: conceptualization, methodology, resources, writing–review and editing, supervision. **Wadir Mario Valentino Marchesiello**: investigation, data curation, writing–original draft. **Micaela Galletta**: investigation, data curation. **Carmelo Coppolino**: investigation, data curation. **Marina Russo**: conceptualization, methodology, writing –review and editing, supervision. **Antonella Buccafuri**: investigation. **Alessia Fazio**: data curation. **Pierluigi Plastina**: methodology, writing–review and editing. **Maurizio Quinto**: methodology, writing–review and editing. **Paola Dugo**: methodology, writing–review and editing. **Luigi Mondello**: methodology, project administration.

## Conflicts of Interest

The authors declare no conflicts of interest.

5

## Supporting information



Supplementary Information

## Data Availability

The data supporting this study's findings are available from the corresponding author, upon reasonable request.
