# Supplementary material for: Elucidating the (lipo)Phenolic Composition of Olive Oil By‐Products and Biomass From Three Different Olea Europaea Cultivars by Liquid Chromatography Coupled to Photodiode Array and Mass Spectrometry
Source: J Sep Sci. 2025 Jun 10;48(6):e70189. doi: 10.1002/jssc.70189 (PMC12149999; doi:10.1002/jssc.70189)
Supplement: Supplementary file 1 — Supplementary Information [file JSSC-48-e70189-s001.docx]

**Table S1**. Matrix effect data (expressed as %) for eriocitrin, naringin and hesperidin in OMWWs, OP, and OLs samples.

| **Compounds** | **OMWW** | | | **OP** | | | **OL** | | |
| --- | --- | --- | --- | --- | --- | --- | --- | --- | --- |
|  | *Roggianella cultivar* | *Coratina cultivar* | *Nocellara cultivar* | *Roggianella cultivar* | *Coratina cultivar* | *Nocellara cultivar* | *Roggianella cultivar* | *Coratina cultivar* | *Nocellara cultivar* |
| Eriocitrin | 123.65 ± 14.33 | 117.15 ± 3.79 | 128.06 ± 7.30 | 109.36 ± 11.83 | 132.92 ± 5.31 | 133.93 ± 1.26 | 71.99 ± 2.46 | 84.75 ± 1.61 | 88.28 ± 3.99 |
| Naringin | 120.03 ± 15.56 | 128.66 ± 3.13 | 138.65 ± 8.96 | 122.49 ± 18.27 | 101.35 ± 3.91 | 111.76 ± 3.33 | 78.59 ± 16.67 | 81.29 ± 1.00 | 92.13 ± 3.77 |
| Hesperidin | 90.34 ± 18.76 | 119.54 ± 16.21 | 130.07 ± 5.79 | 122.10 ± 3.24 | 139.70 ± 17.99 | 133.86 ± 7.28 | 89.11 ± 12.78 | 89.77 ± 9.22 | 79.42 ± 14.59 |
